# Agrobacterial Transformation Enhancement by Improved Competent Cell Preparation and Optimized Electroporation

**DOI:** 10.3390/life13112217

**Published:** 2023-11-17

**Authors:** Xiang Liu, Joseph F. Miceli, Sabrina Patton, Melissa Murray, John Evans, Xiaoping Wei, Pohao Wang

**Affiliations:** Seeds Research, Syngenta Crop Protection LLC, Research Triangle Park, Durham, NC 27709, USAsabrina.patton@syngenta.com (S.P.); melissa.murray@syngenta.com (M.M.); john.evans@syngenta.com (J.E.); xiaoping.wei@syngenta.com (X.W.)

**Keywords:** *Agrobacterium* transformation, competent cell preparation, cell density, plasmid, electroporation, transformation frequency, DMSO

## Abstract

The introduction of plasmids into *Agrobacterium* cells is one of the key steps in the *Agrobacterium*-mediated transformation of plants for gene editing applications. Depending on chromosomal background, some *Agrobacterium* strains exhibit a very low transformation efficiency, which results in a low number of colonies for subsequent screening and thus limits the potential for automated high-throughput transformation processes, especially with low copy or large plasmids. This study demonstrates improvements of transformation frequency by modifying the competent cell preparation process and optimizing electroporation parameters for two *Agrobacterium* strains. The competent cell preparation process was modified by prolonging bacterial growth in the log phase and optimizing the endpoint cell density for cell harvest which resulted in a significant cell yield increase and transformation frequency improvement. Optimization of electroporation by fine-tuning the parameters not only resulted in a 30-fold transformation frequency increase but also revealed a strain-dependent requirement for field strength and electric pulse length. To further improve transformation of a recalcitrant strain, different concentrations of dimethyl sulfoxide (DMSO) in recovery medium were examined. The study revealed an important role of DMSO in transformed cell recovery, with 5% DMSO resulting in the highest transformation frequency. The significant improvements in *Agrobacterium* transformation frequency addressed a critical bottleneck towards establishing a high throughput process.

## 1. Introduction

*Agrobacterium* transformation is one of the key steps in the generation of crop biotech trait events. Agrobacteria are Gram-negative bacteria capable of transferring DNA to plant cells, enabling the delivery of novel genetic material and the creation of new, enhanced crops. New plasmid constructs can be successfully transformed into *Agrobacterium* strains using electroporation, the freeze–thaw method, or triparental mating [1,2,3,4]. However, low transformation frequency is encountered when using large plasmids and with some “recalcitrant” *Agrobacterium* strains. Low transformation frequency may require multiple transformation attempts to obtain the desired *Agrobacterium* strain containing the newly introduced plasmid vector. Low transformation frequency may be caused by several factors: the method used for transformation, competent cell preparation, or biological factors such as the expression of toxic proteins encoded by genes present in the plasmid. Improving transformation frequency will decrease the number of repeated transformations and the associated wasted resources while helping ensure timely and consistent delivery in a research pipeline. In addition, improving transformation frequency may allow for competent cell preparations to be divided into a larger number of usable aliquots from the same starting volume of culture. A larger number of aliquots from each preparation would decrease the number of competent cell preparations performed and decrease the variability in transformation frequency associated with potential lot-to-lot variation.

Electroporation is one of most effective methods for *Agrobacterium* transformation [1,5,6,7]. Electroporation of *Agrobacterium* involves combining electrocompetent cells with the desired plasmid DNA in an electroporation cuvette, subjecting them to a strong electric field with carefully adjusted capacitance and resistance settings. The electric field causes bacterial cell membranes to temporarily become permeable to plasmids [1,8]. The electric field strength is determined by the voltage applied and the gap distance inside the cuvette and is typically 11 kV/cm to 18 kV/cm. The electric pulse length, how long voltage is applied to the sample, is measured as the time constant and determined by the capacitance setting on the instrument and the total resistance (instrument internal resistance and sample resistance) [9,10,11,12]. Typical time constants used for *Agrobacterium* transformation are 4–8 ms (millisecond). After the electrical pulse is applied, the cells are incubated in non-selective recovery media to allow the cells to express antibiotic resistance genes prior to performing selection on agar media for isolation of individual colonies.

Preparation of electrocompetent cells requires culturing active bacterial cells, washing away excess salts, concentrating the cells, and cryogenically preserving them [11,13,14,15]. Actively growing cells are harvested from liquid culture to ensure that cells are ready to express resistance genes upon transformation and subsequent selection on antibiotic media. Higher culture densities allow for a greater number of aliquots to be prepared while ensuring there are enough colonies after transformation for successful screening. Excess salts are washed from the cells to avoid short circuiting during electroporation, a process where the electric field does not form as electrons flow through the bacterial sample due to low resistance. After washing, cells are resuspended in a small volume of buffer to concentrate them. The buffer used for resuspension, a glycerol mixture, acts as a cryoprotectant and provides a high resistance to facilitate subsequent electroporation.

Disarmed *Agrobacterium* LBA4404 and Chry5 strains with modified helper plasmids have been used for achieving high efficiency transformation in soybean and maize, respectively [16,17,18]. The Chry5 strains often demonstrate faster growth, but much lower transformation frequencies than the LBA4404 strains, routinely resulting in a limited number of colonies after antibiotic selection. Improving transformation frequency for Chry5 strains will greatly improve the capability to test multiple clones from each transformation. Additionally, a higher transformation frequency will mean that competent cells in each lot can be separated into a larger number of aliquots, decreasing the number of lots prepared and the associated cost of validating them.

This work focuses on testing three hypotheses for improving our transformation process. First, an optimal time during log phase growth of cultures can be identified for harvesting competent cells to increase cell yields and competency. Second, the field strength and time constant settings can be optimized for electroporation to improve transformation frequency. Third, optimizing the concentration of DMSO in recovery media can increase transformation frequency of recalcitrant *Agrobacterium* strains as shown for mammalian cell electroporation [19].

Based on these hypotheses, we varied electroporation settings for Chry5 and LBA4404 strains by fine-tuning parameters (voltage, gap distance, capacitance, and resistance) and modified the competent cell preparation process to improve cell yield and competency. For enhancing transformation frequency, we varied cell densities at the time of culture harvest and the addition of DMSO to recovery media. These parameters were optimized for multiple *Agrobacterium* strains with different plasmids known to demonstrate either high or low transformation frequency.

## 2. Materials and Methods

### 2.1. Plasmids and Agrobacterial Strains

Five binary plasmid vectors were used to test transformation frequency, including 23093 (11.4 kb), 26425 (22.2 kb), 25641 (23.7 kb), 26246 (22.0 kb), and 26334 (24.5 kb). These binary vectors contain a spectinomycin resistance marker gene (aadA) in the vector backbone [20]. Plasmids were replicated in *E. coli* DH5alpha using Luria-Bertani (LB) medium supplemented with 50 mg/L spectinomycin. All *E. coli* cultures were incubated at 37 °C. Plasmid DNA was prepared using the PureYield plasmid miniprep system (Promega A1223) following the manufacturer’s instructions. The plasmid sequences were validated by NGS sequencing.

Two *Agrobacterium* strains, Chry5d3 recA- for soybean transformation and LBA4404 (17740) recA- for maize transformation (abbreviated as LBA4404 and Chry5 in rest of this article) [16,17], were used in this study. Agrobacterial cultures were isolated on YP plates (1% Bacto-yeast extract, 2% Bacto-peptone, and 2% Bacto-agar) supplemented with appropriate antibiotics. All *Agrobacterium* cultures were incubated at 28 °C.

### 2.2. Electro-Agrobacterium Transformation

Electroporation was performed with a BTX Precision Pulse ECM630 electroporator with VWR Signature™ Disposable Electroporation Cuvettes (89047-206/89047-208). An amount of 250 ng of plasmid DNA and 40 μL of competent cells were mixed for each transformation. Transformed cells were then allowed to recover in 500 μL of YP medium for 3 h at 28 °C with shaking (for Chry5) or without shaking (for LBA4404).

To optimize the frequency of *Agrobacterium* electroporation, various parameters, field strength (voltage/cuvette gap distance) and time constant (resistance × capacitance), were fine-tuned and examined using appropriate plasmids for each *Agrobacterium* strain. Based on preliminary examinations, nine settings were further selected for evaluating the transformation frequency of Chry5. Voltage settings of 2.2, 2.5, and 2.8 kV were tested along with 150 or 200 ohms of resistance and a capacitance of either 50 or 35 μF with three plasmids (23039, 25641, and 26425, Table 1). Based on preliminary examinations, eight settings were chosen for evaluating the transformation frequency of LBA4404. Voltage settings of 1.8, 2.5, and 2.8 kV, a capacitance of either 25 or 50 μF, and cuvette gap sizes of 0.2 or 0.1 cm were tested with two plasmids (26246 and 26334, Table 2). For the optimization of LBA4404, the time constant was varied only by changing capacitance, and the resistance was kept constant.

### 2.3. Transformation Frequency Calculations

After a 3 h incubation, 100 μL (for Chry5) or 50 μL (for LBA4404) of cell suspension was plated on YP plates containing appropriate antibiotics and incubated at 28 °C for up to three days (for Chry5) or four days (for LBA4404). For comparative analysis, the frequency of each transformation was calculated by counting the number of colonies formed and presented as colony-forming units (CFUs) per microgram (μg) DNA used during electroporation. To eliminate the difference between plasmids, the frequency for each sample was normalized by using the average transformation frequency of all plasmids divided by the average transformation frequency of the associated plasmid. The data analysis was conducted using Microsoft Excel (Version 2302).

### 2.4. DMSO Test in Recovery Medium

The role of DMSO in the recovery medium was investigated for Chry5 transformation. Various DMSO concentrations were compared multiple times. A small range of lower concentrations of DMSO (0–3%) were tested first. Then, a broader range of DMSO concentrations (0–9%) were tested for further optimization. The optimized electroporation settings (2.8 kV/cm, 150 ohms, and 50 μF) were applied for all DMSO tests. Transformed cells were recovered in 500 μL of YP medium supplemented with the appropriate concentration of DMSO and incubated at 28 °C for 3 h. Following the recovery period, 100 μL (high efficiency plasmid) and 200 μL (low efficiency plasmid) of the recovered cells were plated on YP plates supplemented with 100 mg/L of ampicillin and 500 mg/L of spectinomycin. The plates were incubated at 28 °C for three days. The colonies obtained from each treatment were counted and presented as CFU/μg. The data analysis was conducted using Microsoft Excel.

### 2.5. Competent Cell Preparation and Validation

#### 2.5.1. Agrobacterium Initiation and Inoculation

*Agrobacterium* Chry5 was inoculated with a single colony from a freshly streaked plate in 1 mL of YP medium containing 100 mg/L of ampicillin. This culture was incubated at 28 °C with shaking at 300 rpm for eight hours and then was transferred into a 1000 mL flask containing 200 mL of YP medium supplemented with 100 mg/L of ampicillin and 0.1 mM EDTA for overnight growth at the same condition.

After 15 h overnight incubation, cell density was measured using a spectrophotometer (Eppendorf^®^, Hamburg, Germany). When the OD_600_ reached 2.0, varied amounts of the culture were transferred to fresh YP medium (room temperature) supplemented with 100 mg/L of ampicillin and 0.1 mM EDTA to extend the growth and optimize the endpoint cell density (Table 3). All six resulting subcultures were incubated at 28 °C for an additional four hours (three hours in the preliminary experiment).

#### 2.5.2. Competent Cell Preparation

After the extended culture time, cell densities were measured, and immediately each subculture was transferred into 250 mL ice cold centrifuge tubes and incubated on ice for 20–30 min. After the ice incubation, the cells were pelleted by spinning at 6000 RPM for 15 min at 4 °C in a refrigerated high-speed centrifuge (Avanto JXN-26, Beckman Coulter, Indianapolis, IN, USA) and the supernatant was poured off. The pellet from each subculture was washed three times with 200 mL, 100 mL, and 8 mL of ice-cold 10% glycerol. After each wash, the cells were pelleted using the above conditions. At the end of the three wash cycles, the supernatant was poured off, and each cell pellet was resuspended in 2 mL of ice-cold 10% glycerol. The cell suspensions were then aliquoted (40 μL each) into chilled sterile 1.1 mL mini tubes (Axygen™ Mini Tube System, Corning, Salt Lake City, UT, USA) on dry ice. The cells were stored at −80 °C for transformation tests.

#### 2.5.3. Cell Validation and Antibiotics Resistance Tests

The source Chry5 glycerol stock was validated by PCR. In this study, the newly prepared competent cells were further validated by antibiotics tests. An amount of 250 ng of plasmid DNA was introduced into 40 μL of competent cells by electroporation with the strain-dependent optimal settings (see above). The transformed cells were recovered in 500 μL of YP medium containing 3% DMSO for 3 h and then plated on YP plates supplemented with 100 mg/L of ampicillin for three days of incubation at 28 °C. The numbers of resulting colonies from each transformation were counted and presented as CFU/μg for comparative analysis. The data analysis was conducted using Microsoft Excel. For negative controls, the null transformation and recovery was conducted at the same conditions with no plasmid DNA added. The recovered cells were plated on a series of YP media containing different antibiotics (Table 4).

## 3. Results

### 3.1. Strain-Dependent Optimization of Time Constant and Field Strength

**Figure 1:**
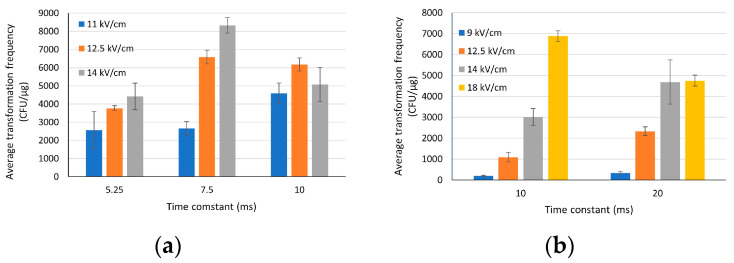
Electroporation setting optimization. Field strength is displayed by the color of the column. (**a**) Average transformation frequency of three different plasmids for the Chry5 strain across nine electroporation settings. (**b**) Average transformation frequency of two different plasmids for the LBA4404 strain across eight electroporation settings. Error bar represents standard deviation (*n* = 5 for Chry5, *n* = 2 for LBA4404). The data analysis was conducted using Microsoft Excel.

To improve transformation frequency, electroporation parameters, field strength (voltage/cuvette gap) and time constant (resistance x capacitance), were optimized for two *Agrobacterium* strains, Chry5 and LBA4404. The transformation frequency of nine selected settings for the Chry5 strain were evaluated using three different plasmids (Table 1). Figure 1a presents the transformation frequency (CFU/μg) derived from different combinations of field strength and time constant for the Chry5 strain. At low time constants (5.25 ms and 7.5 ms), the higher field strength resulted in higher transformation frequency. However, at a high time constant (10 ms), the transformation frequency showed a negative correlation with field strength. The combination of a field strength of 14 kV/cm with a time constant of 7.5 ms resulted in the highest transformation frequency for the Chry5 strain.

The transformation frequency for LBA4404 was evaluated using two different constructs (Table 2). Based on preliminary experiments, the time constant was varied only by changing capacitance, and resistance was kept constant. At both time constants, higher field strength resulted in higher transformation frequency (Figure 1b). However, the frequency dropped when high field strength was applied with a longer time constant (18 kV/cm × 20 ms). The highest frequency was achieved at the combination of 18 kV/cm for the field strength and 10 ms for the time constant. For the LBA4404 strain, the best transformation settings for both the field strength and time constant are higher than those for the Chry5 strain. This disparity may arise from the varying sensitivity of each strain’s cell membrane to the transient electric pulse during electroporation.

### 3.2. DMSO Concentration Plays an Important Role in Cell Recovery of the Chry5 Strain

**Figure 2:**
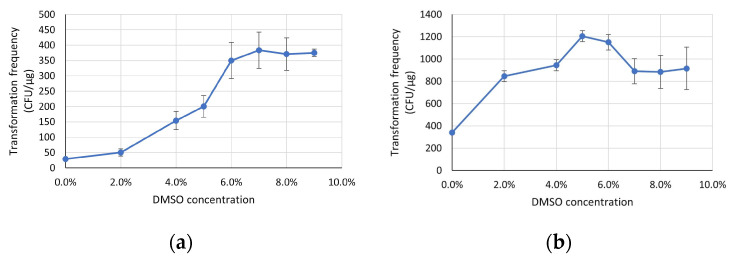
Effect of DMSO concentration in recovery media on transformation frequency of the Chry5 strain using plasmid 25641 ((**a**), *n* = 2) and plasmid 26425 ((**b**), *n* = 2). Frequency presented as CFU/μg DNA. Error bars represent standard deviation. The data analysis was conducted using Microsoft Excel.

Chry5 has a low transformation frequency compared to other strains, such as LBA4404 and EHA101. To increase its transformation frequency, various concentrations of DMSO in the recovery medium were examined. Our preliminary data with comparisons of low DMSO concentrations suggested that DMSO played an important role in electro-transformation recovery (Figure S1). Based on the preliminary data, transformation frequencies of DMSO concentrations (0–9%) were evaluated with two different plasmids (Figure 2). In the test with plasmid 25641, the frequency positively correlated with DMSO concentration up to 6%, which showed about a 10× increase compared to the frequency with no DMSO in the recovery medium (Figure 2a). In the test with another plasmid, 26425, the transformation frequency positively correlated with DMSO concentration until 5%, with a frequency increase of more than 3× in comparison with transformations with no DMSO in the recovery medium (Figure 2b). Transformation frequency decreased slightly when DMSO concentration was higher than 6%.

### 3.3. Chry5 Competent Cell Prep and Transformation Improvement

Preliminary testing indicated that the Chry5 culture growth log phase occurred between 0.3 and 2.0 OD_600_ (Figure S2). To increase the cell yield and enhance cell competency, the culture growth of the log phase was extended for 3 h by providing room temperature fresh media (and increasing culture volume) after 15 h of overnight incubation. Meanwhile, six subcultures were made with a series dilution from the same overnight culture to optimize the endpoint cell densities (Table 3 and Table 5). After the extended incubation, the cell densities of subcultures ranged from 0.41 to 1.36 OD_600_ (Table 5, optimization I). Competent cell aliquots were prepared from these subcultures as described in the methods section. The transformation frequencies of the competent cells were evaluated with two plasmids and presented in a range of 0–824 CFU/μg for plasmid 25641 and a range of 128–3792 CFU/μg for plasmid 26425. The results demonstrated that higher a transformation frequency resulted from a higher cell density.

**Figure 3:**
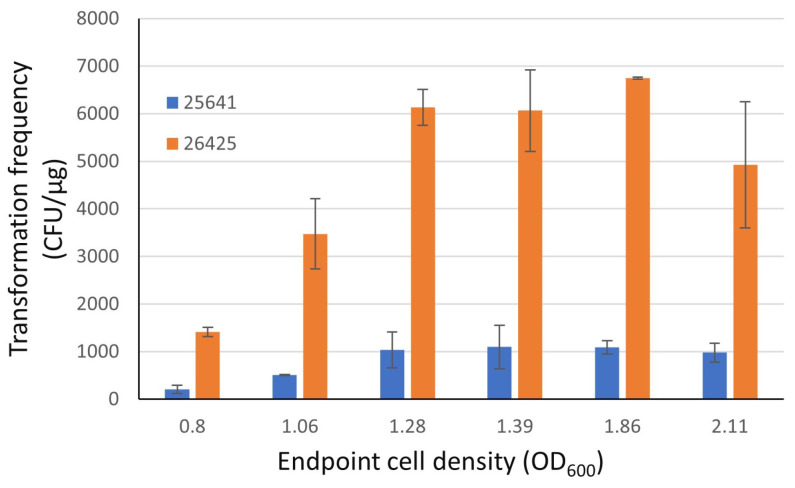
Effect of endpoint cell density on transformation frequency during extended log phase growth of competent cell preparation. The endpoint cell density was measured after four-hour extension (*n* = 2). After competent cell preparation and aliquoting, transformation frequency was evaluated with two plasmids, presented as CFU/μg DNA. Error bar represents standard deviation. The data analysis was conducted using Microsoft Excel.

Based on the preliminary results, it was decided to extend the incubation time from three hours to four hours for six subcultures in the final experiment. At the end of the four-hour extended incubation, cells were harvested with an OD_600_ ranging from 0.80 to 2.11 (Table 5, Optimization II) and a transformation frequency ranging from 207 to 1097 CFU/μg with plasmid 25641 and 1414 to 6750 CFU/μg with plasmid 26425 (Figure 3). The highest numbers of colony counts were achieved at cell densities between 1.28 and 1.86 OD_600_.

The transformation frequency of cells obtained using the extended growth protocol with an OD_600_ of 1.28–1.39 was compared with existing competent cell lots derived from a protocol without log phase extension via fresh media amendment. The optimized extended growth protocol resulted in about a 109% increase in transformation frequency (Figure S3).

## 4. Discussion

Bacterial growth, an increase in population via cell division, exhibits four phases: lag, log, stationery, and death phase. The cell numbers increase logarithmically in the log phase until nutrients are depleted, or toxic products accumulate, which results in slow growth and cell death [21,22]. In this study, the log phase of bacterial growth was prolonged by supplementing fresh media to sustain rapid growth for additional hours. As the cell number increased rapidly during the extended log phase, the ratio of dead cells to live cells could be significantly reduced. Therefore, the increased transformation frequency in the current modified protocol may be attributed to both enhanced cell competency and improved yield. An additional benefit of log phase extension is the convenience and flexibility for reaching the optimal cell density for cell harvesting. Because this extension can be performed in the daytime, overgrowth or insufficient growth caused by overnight incubation can be avoided.

An optimal cell density for competent cell preparation is critical. However, different endpoint cell densities were suggested in different protocols [5,14,23]. In the current study, a higher transformation frequency was observed in cultures with an OD_600_ of 1.2–1.8, which focused on the improvement of a low-frequency *Agrobacterium* strain. For comparison, we also optimized the endpoint cell density for LBA4404 competent cell preparation, in which the same YP medium but different dilution and growth time parameters were used. The highest transformation frequency was achieved from the culture with an OD_600_ of 0.8 (Figure S4). This suggests that optimal endpoint cell density may be strain- and growth condition-dependent. In addition, we observed inconsistencies in OD_600_ readings between spectrophotometers of different brands. While this discrepancy may stem from various factors, the ultimate consequence is that OD_600_ readings may lack consistency across different instruments and consequently between different laboratories. Therefore, optimizing the growth condition and endpoint cell density for each specific strain in each laboratory is likely necessary.

Electroporation is one of the most frequently employed techniques for introducing foreign DNA into bacteria [1]. Since its first publication, electroporation has been widely applied across disciplines to introduce DNA into cells [5,6,24]. It takes advantage of large electric fields to temporarily modify the cell membrane’s permeability [1]. The electric pulses enable the formation of temporary pores in the cell membrane to allow DNA molecules to enter the cells. The current study demonstrated higher transformation frequencies derived from higher electric field strength (Figure 1). However, higher field strength did not improve transformation frequency when the duration of electric pulses extended beyond a critical threshold. This may be the result of irreparable cellular damage due to excessively high field strength or long pulses [1,25]. Furthermore, the optimal settings differed between two *Agrobacterium* strains. This implies different tolerances for field strength and pulse length between these two strains. The strain-dependent settings also suggested the importance of the balance of field strength and time constants in successful electroporation.

To optimize the time constant for LBA4404, the effect of time constants (10 ms, 15 ms, and 20 ms) on transformation frequency was also tested at the same field strength, 14 kV/cm. The results still support the conclusion that the best settings are 14kV/cm for 10 ms for the LBA4404 strain. It should be noted that all electroporators have a maximum voltage output. For some electroporators to provide these high field strengths, using cuvettes with smaller gaps may be required. Considering the many different possible experimental conditions, such as laboratory equipment, culture medium, bacterial strains, cell quality, and recovery environment, the optimal electroporation setting may not be identical across different laboratories.

DMSO is a well-known membrane permeation enhancer [26,27]. It can induce water pores in dipalmitoyl-phosphatidylcholine bilayers and causes the membrane to become floppier, which would enhance permeability and facilitate membrane fusion [28]. The current study suggested that DMSO plays an important role in transformation recovery after electroporation. Increasing DMSO concentrations (0–6%) in the recovery medium significantly enhances the transformation frequency of a recalcitrant *Agrobacterium* strain, Chry5. Similarly, the transformation enhancement by DMSO was also reported in eukaryote cell transformations [19,29]. Reporter gene activity increased up to eightfold after incubation of mammalian cells with DMSO after electroporation [19]. Plasmid DNA was directly delivered into intact plant cells by electroporation with a short exposure of the cells to 2% DMSO prior to plasmolysis [29]. In one aspect, high DMSO concentration allowed for large molecules to pass through membranes while intermediate concentrations only enabled water and calcium to enter the cells [30]. On the other hand, for transformed cells to exhibit resistance and recover from the damage after electroporation, it is crucial for them to express the antibiotic resistance gene [25,31]. Therefore, the observed enhancement in bacterial transformation frequency, achieved by adding DMSO to the recovery medium, is likely associated with both an increased movement of plasmid transfer and improved uptake of essential nutrients.

## Figures and Tables

**Table 1:** Settings of Chry5 optimization.

Setting	Chryt1	Chryt2	Chryt3	Chryt4	Chryt5	Chryt6	Chryt9	Chryt10	Chryt11
Voltage (kV)	2.2	2.5	2.8	2.2	2.5	2.8	2.2	2.5	2.8
Resistance (ohms)	150	150	150	200	200	200	150	150	150
Capacitance (uf)	50	50	50	50	50	50	35	35	35
Gap (cm)	0.2	0.2	0.2	0.2	0.2	0.2	0.2	0.2	0.2
Field strength (kV/cm)	11	12.5	14	11	12.5	14	11	12.5	14
Time constant (ms)	7.5	7.5	7.5	10	10	10	5.25	5.25	5.25

**Table 2:** Settings of LBA4404 optimization.

Setting	LBAt1	LBAt2	LBAt3	LBAt4	LBAt5	LBAt6	LBAt7	LBAt8
Voltage (kV)	1.8	2.5	2.8	1.8	2.5	2.8	1.8	1.8
Resistance (ohms)	400	400	400	400	400	400	400	400
Capacitance (uf)	25	25	25	50	50	50	25	50
Gap (cm)	0.2	0.2	0.2	0.2	0.2	0.2	0.1	0.1
Field strength (kV/cm)	9	12.5	14	9	12.5	14	18	18
Time constant (ms)	10	10	10	20	20	20	10	20

**Table 3:** Subculture preparation from series dilutions of overnight culture.

Subculture	Fresh YP Medium (mL)	Overnight Culture (mL)	Total Volume (mL)
A	190	10	200
B	185	15	200
C	180	20	200
D	175	25	200
E	170	30	200
F	165	35	200

**Table 4:** YP media for antibiotics test.

Antibiotics	Ampicillin	Tetracycline	Kanamycin	Spectinomycin	Gentamicin
Con. (µg/mL)	100	10	50	500	25

**Table 5:** Endpoint cell density.

Subculture	Optimization I		Optimization II	
	Extension (Hour)	OD_600_	Extension (Hour)	OD_600_
A	3	0.41	4	0.80
B	3	0.62	4	1.06
C	3	0.80	4	1.28
D	3	1.02	4	1.39
E	3	1.20	4	1.86
F	3	1.36	4	2.11

## Data Availability

Data are contained within the article and supplementary materials.

## Supplementary Materials

The following supporting information can be downloaded at https://www.mdpi.com/article/10.3390/life13112217/s1, Figure S1: Different concentrations of dimethyl sulfoxide (DMSO) were applied in recovery medium; Figure S2: Chry5 culture growth observations; Figure S3: Transformation frequency comparison; Figure S4: Optimization of endpoint cell density for LBA4404 competent cell preparation.
