# Supplementary material for: Agrobacterial Transformation Enhancement by Improved Competent Cell Preparation and Optimized Electroporation
Source: Life (Basel). 2023 Nov 17;13(11):2217. doi: 10.3390/life13112217 (PMC10671908; doi:10.3390/life13112217)
Supplement: Supplementary file 1 [file life-13-02217-s001.zip › Supplementary caption.pdf]

Figure S1. Different concentrations of dimethyl sulfoxide (DMSO) were applied in recovery medium. The frequency of each transformation was evaluated and calculated by counting the number of colonies and presented as CFU/μg DNA; Figure S2. Chry5 culture growth observations. Two Chry5 colonies (A and B) were inoculated in different volumes (3 mL and 6 mL) of YP medium and incubated at 28°C. After overnight incubation, cell densities (OD600) were measured hourly; Figure S3. Transformation frequency comparison. The transformation frequency of cells derived from cultures with OD600 of 1.28 and 1.39 was compared with existing cells derived from an existing protocol without log phase extension. Transformation frequency was evaluated with plasmid 26425 and calculated by counting the number of colonies and presented as CFU/μg DNA. Error bar represents standard deviation. The data analysis was conducted using Microsoft Excel; Figure S4. Optimization of endpoint cell density for LBA4404 competent cell preparation. Transformation frequencies were evaluated with two plasmids (26246 and 26334) and calculated by counting the number of the colonies and presented as CFU/μg DNA. Error bar represents standard deviation. The data analysis was conducted using Microsoft Excel.
