# Supplementary figures and images for: Agrobacterial Transformation Enhancement by Improved Competent Cell Preparation and Optimized Electroporation
Source: Life (Basel). 2023 Nov 17;13(11):2217. doi: 10.3390/life13112217 (PMC10671908; doi:10.3390/life13112217)

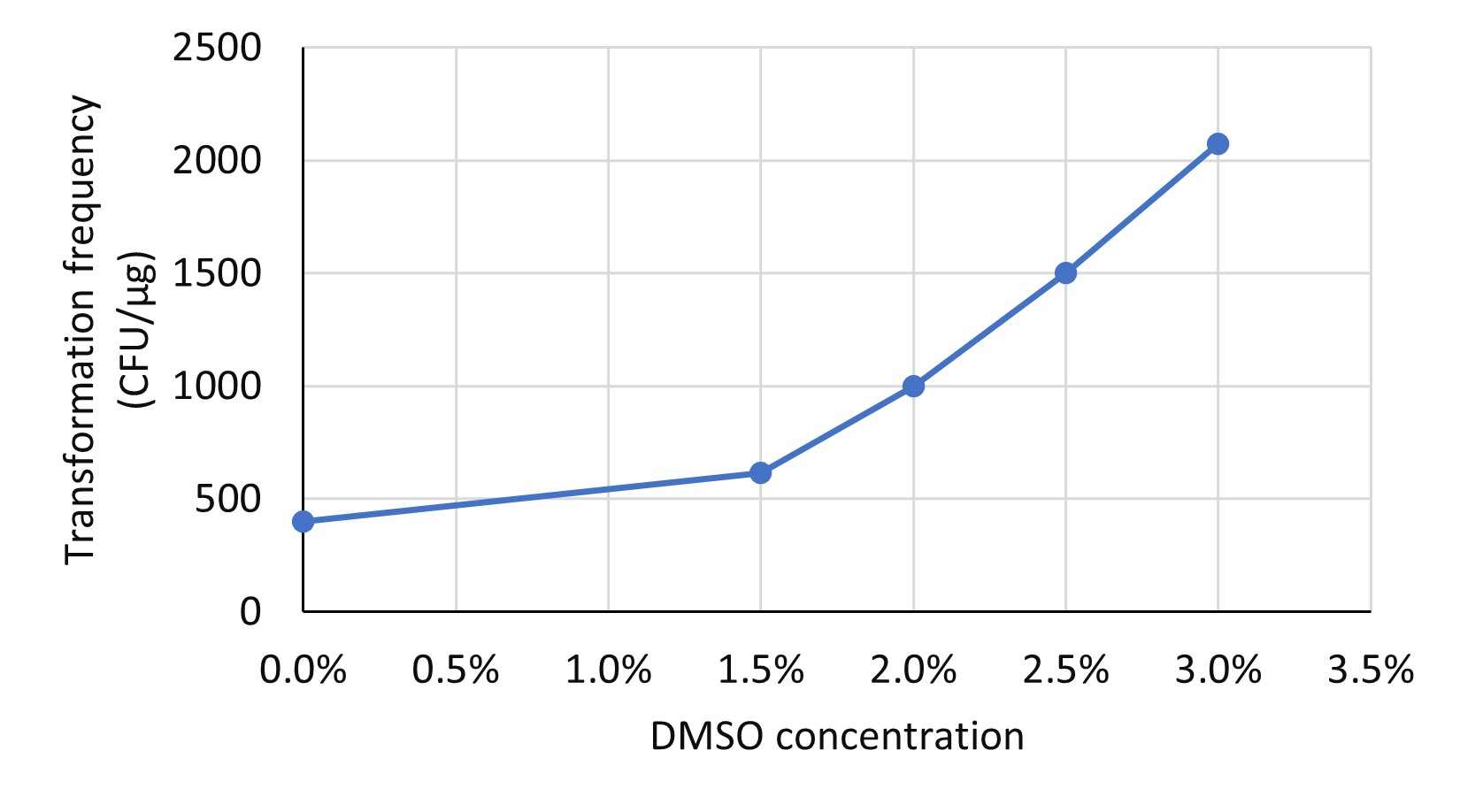

Supplement: Supplementary file 1 [file life-13-02217-s001.zip › Picture S1.jpg]

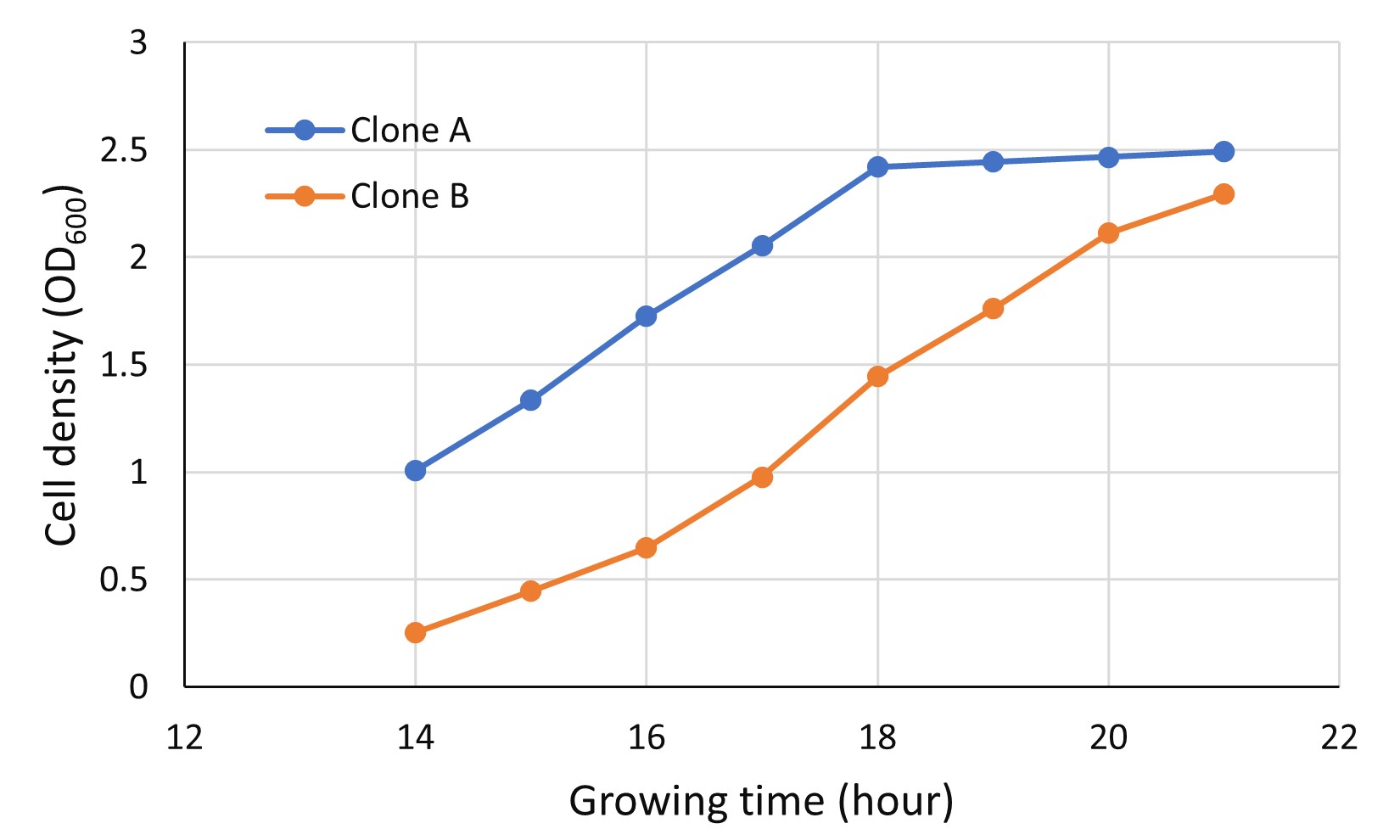

Supplement: Supplementary file 1 [file life-13-02217-s001.zip › Picture S2.jpg]

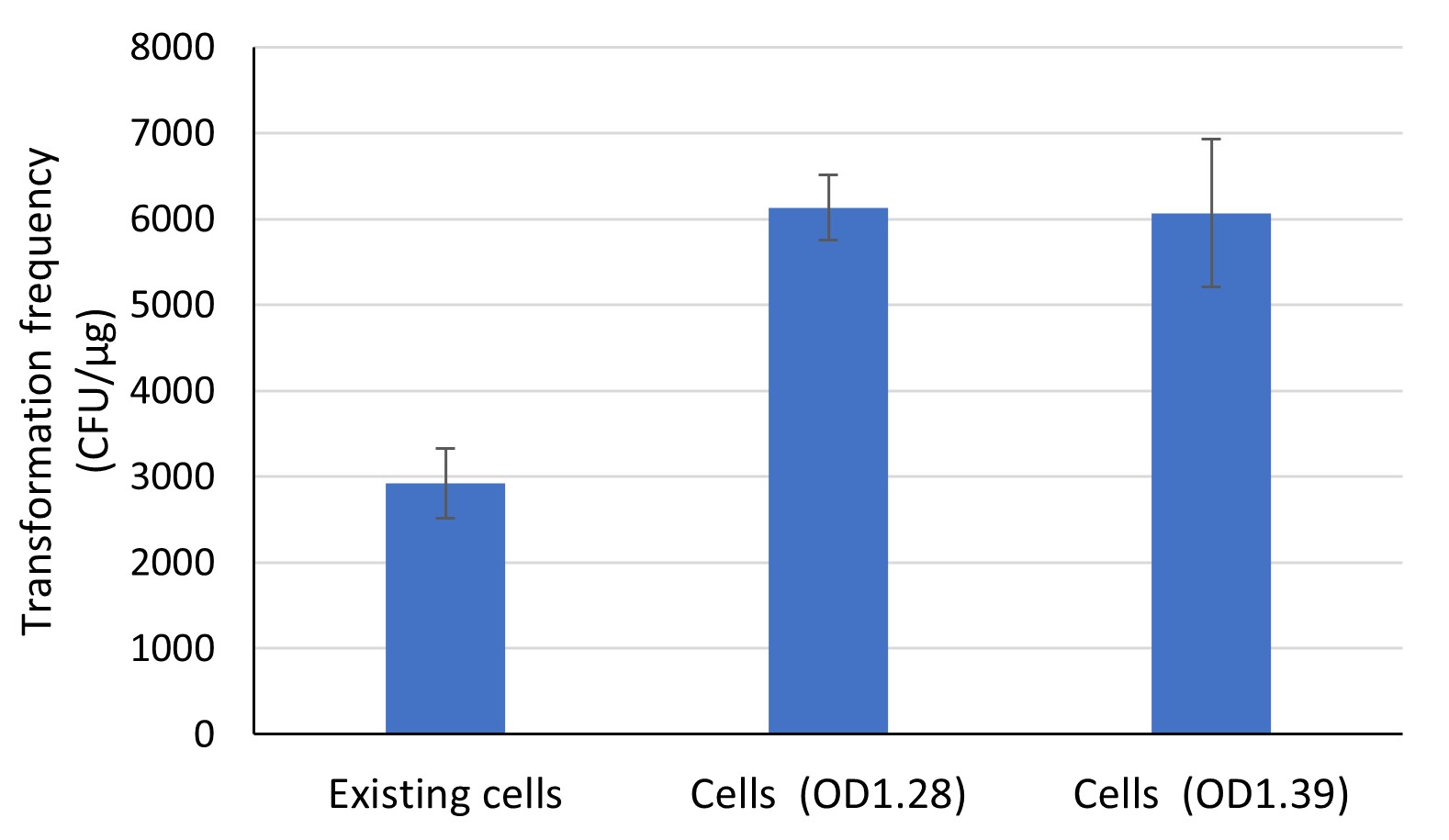

Supplement: Supplementary file 1 [file life-13-02217-s001.zip › Picture S3.jpg]

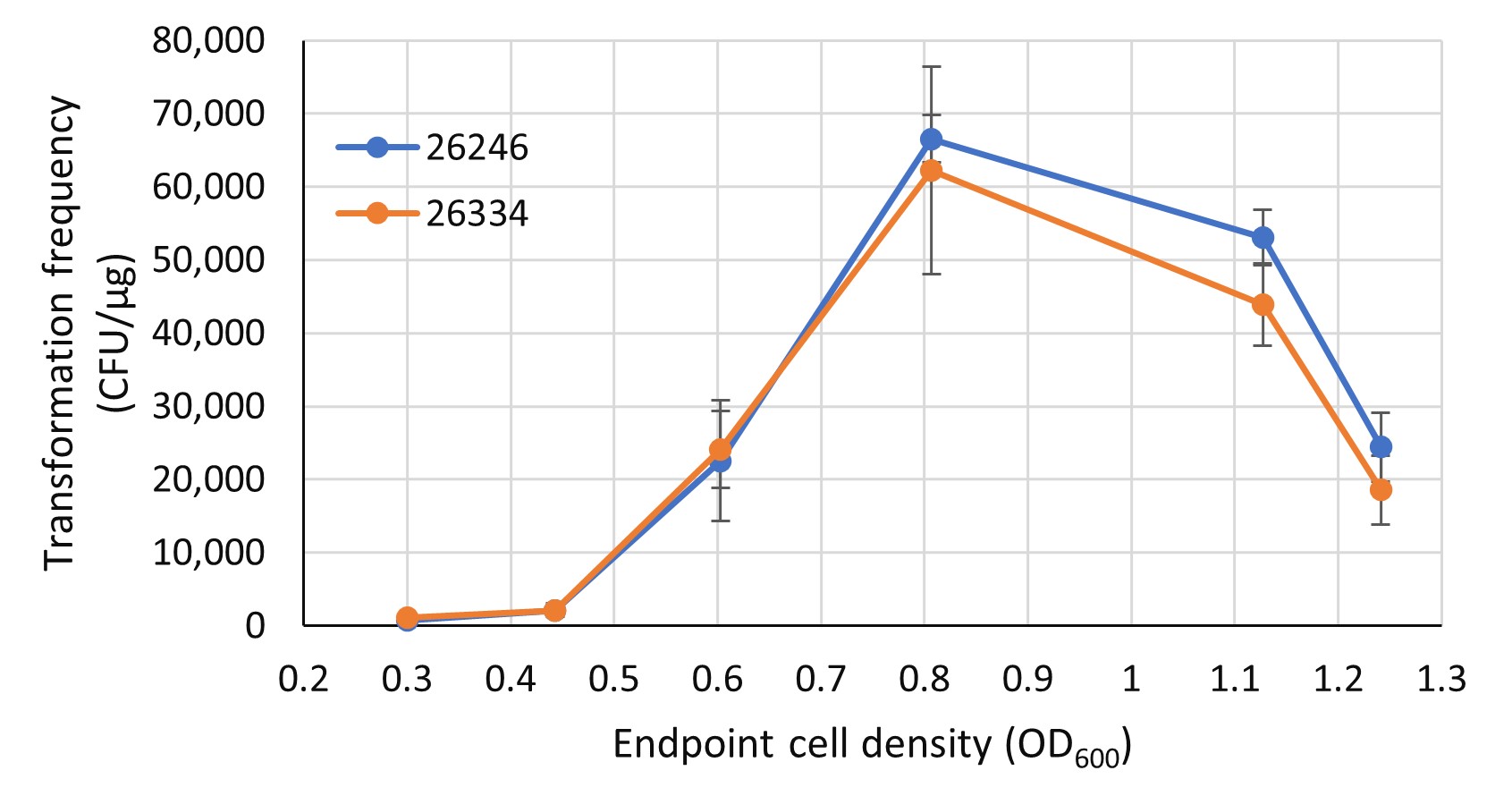

Supplement: Supplementary file 1 [file life-13-02217-s001.zip › Picture S4.jpg]
